# Prevalence of bluetongue virus disease in a small ruminant population in Kalat, Balochistan, Pakistan

**DOI:** 10.14202/vetworld.2024.1966-1971

**Published:** 2024-09-01

**Authors:** Shafiq Ahmad, Muhammad Shafee, Abdul Razzaq, Farhad Badshah, Naimat Ullah Khan, Eliana Ibáñez-Arancibia, Patricio R. De los RíosEscalante, Hafiz Muhammad Arif, Abid Hussain

**Affiliations:** 1 Balochistan Agriculture Research and Development Center (BARDC), Quetta, Pakistan; 2 Center for Advanced Studies in Vaccinology and Biotechnology(CASVAB), University of Balochistan, Quetta, Pakistan; 3 Animal Sciences Division, Pakistan Agricultural Research Council, Islamabad, Pakistan; 4 Department of Zoology, Abdul Wali Khan University Mardan, Khyber Pakhtunkhwa Pakistan; 5 State Key Laboratory of Animal Biotech Breeding, Institute of Animal Sciences, Chinese Academy of Agricultural Sciences, Beijing 100193, China; 6 Shenzhen Branch, Guangdong Laboratory of Lingnan Modern Agriculture, Key Laboratory of Livestock and Poultry Multi-Omics of MARA, Agricultural Genomics Institute at Shenzhen, Chinese Academy of Agricultural Sciences, Shenzhen, 518000, China; 7 College of Veterinary Sciences and Animal Husbandry, Abdul Wali Khan University Mardan, Khyber Pakhtunkhwa, Pakistan; 8 PhD Program in Sciences mentioning Applied Molecular and Cell Biology, La Frontera University, Temuco, Chile; 9 Department of Chemical Engineering, Laboratory of Engineering, Biotechnology and Applied Biochemistry, Faculty of Engineering and Science, La Frontera University, Temuco, Chile; 10 Department of Biological and Chemical Sciences, Faculty of Natural Resources, Catholic University of Temuco, Temuco, Chile; 11 Nucleus of Environmental Sciences, Faculty of Natural Resources, Catholic University of Temuco, Temuco, Chile

**Keywords:** antibodies, Balochistan, bluetongue virus, competitive enzyme-linked immunosorbent assay, Kalat

## Abstract

**Background and Aim:**

Bluetongue is a vector-borne, emerging disease that poses a severe threat to most domesticated animals. A cross-sectional study was conducted to estimate the prevalence of bluetongue virus (BTV) disease in apparently healthy sheep and goats in Kalat, Balochistan.

**Materials and Methods:**

A total of 270 serum samples (sheep: 150 and goat: 120) were collected and screened for the detection of anti-BTV antibodies using a competitive enzyme-linked immunosorbent assay. The data regarding different contributory risk factors were also collected using a predesigned questionnaire.

**Results:**

It revealed that overall, 27.4% (74/270, 95% confidence interval, χ^2^ = 1.71, p = 0.12) prevalence in both sheep and goat populations. The highest prevalence of 47% (32/68) was recorded in Surab city with the lowest prevalence of 15.49% (11/71) in the Manguchar area. In contrast, in Kalat 28.1% (9/32), Daan area 24% (12/50), and Marap area 22.44% (11/49), seropositivity was recorded. Upon sex bases, antibodies were almost equally found in both male 28.57% (8/28) and female 27.27% (66/242) animal populations. Moreover, all four breeds (Balochi, Khurasani, Lehri, and Rakhshani) were equally and potentially seropositive. The Khurasani breed was the most susceptible to 34.69% (17/49), followed by the Balochi breed, 45/145 (31%) seropositivity. The prevalence of BTV was 16.66% (1/6) in Rakhshani breed and 15.71% (11/70) in Lehri breed., Ticks were found in almost 21% of animals, while 93% of animals were reared on open grazing in rangelands.

**Conclusion:**

This study clearly indicates widespread BTV infection in small ruminants in the study area that may pose serious threats to livestock farming. Further extensive studies are recommended to study the prevalence of disease in different agroecological zones of the province. This also warns the high-ups to manage concrete efforts to eradicate and control the disease in the area.

## Introduction

Bluetongue virus (BTV) is one of the primary causes of economic losses to farmers and has been included in the World Organization for Animal Health’s (WOAH) lists of notifiable diseases [1]. It is an arthropod-borne non-contagious disease that mostly occurs in sheep and some other wild and domestic ruminants [2]. The disease is caused by BTV that belongs to a member of the genus *Orbivirus* [3]. BTV is spread by different species of *Culicoides* (biting midges), which are most abundant and active in humid and hot climates. Among 1400, only 20 species of *Culicoides* midges are documented to be responsible for transmitting the disease [4]. Globally, up to 3 billion US$ economic losses have been reported [5]. Pathogenesis usually involves vascular endothelial damage, resulting in hyperemia and gynecological problems [3]. Clinical manifestations include excessive salivation, fever, conjunctival and nasal discharge, cyanosis of the tongue, generalized hyperemia, and facial edema [6]. Different factors such as immune status, age, breed, nutritional status, environmental stress, and the infecting viral strain are responsible for the severity of the disease [7, 8]. In goats and cattle, infection is usually asymptomatic, but sheep are more commonly infected [9]. It is a self-limiting disease in the acute stage, and so far, 27 different serotypes have been recognized throughout the World, including the Indo-Pak subcontinent, China, Iran, and Afghanistan. Recently, in Pakistan (Balochistan), serotypes 8 and 9 have been recognized using real-time polymerase chain reaction [10]. Various techniques, such as the hemagglutination-inhibition test, serum neutralization test, agar gel diffusion, and enzyme-linked immunosorbent assay (ELISA), are employed to detect BTV infection [3]. The competitive-ELISA (c-ELISA) is a rapid diagnostic assay that can widely be used to detect immunoglobulins in the very early stages of infection and is also recommended by WOAH for international trade [11, 12]. The semi-arid climate, low/poor quality feed, meager animal husbandry practices, low-cost infrastructure, and nomadic culture are some of the major constraints to the local livestock sector, leading to high economic losses throughout the province [13].

Balochistan, the largest province in Pakistan, has over 50% of its population relying directly on agriculture and livestock rearing. The preference for small ruminants is influenced by the inhabitants’ transhumant, nomadic lifestyle and various geographical factors within the province. The absence of diagnostic facilities in these remote rural areas leads to significant direct and indirect economic losses for farmers. This study aimed to estimate the prevalence and identify contributory risk factors of BTV in sheep and goat herds in Kalat, Balochistan, Pakistan.

## Materials and Methods

### Ethical approval and Informed consent

The institutional ethical committee of the Center for Advanced Studies in Vaccinology and Biotechnology (CASVAB), University of Balochistan, Quetta, approved this study (Approval no. CASVAB/04/18). Samples were collected without any harm to animals and with the verbal consent of farmers strictly following the established animal welfare guidelines.

### Study period and location

**Figure-1:**
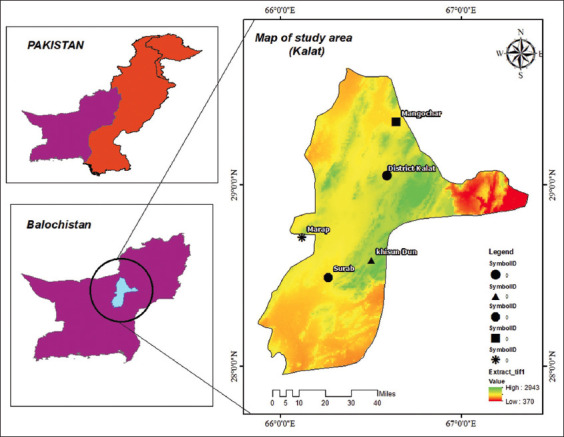
Geographical location of district Kalat, Balochistan. Stars indicate blood sampling sites [Source: The map was generated using ArcGIS 10.8].

The study was conducted from April to July-2018. For this study, sheep and goat populations from five diverse epidemiological units, Surab (Sikandarabad), Kalat, Dan, Manguchar, and Marap, within Baluchistan province, were selected (Figure-1). District Kalat, is situated between longitudes 65°49’50” and 67°27’56” East and latitudes 27°55’55” and 29°37’43” North, with moderate summer and mild to severe cold in winter season. Over 70% of its inhabitants depend on livestock rearing, particularly sheep and goats, for their livelihood [14].

### Sample collection

Blood samples were collected from 270 small ruminants, including Balochi and Khurasani sheep breeds (n = 150) and goat breeds, including Lhari and Rukhshani (n = 120), which were randomly selected and previously unvaccinated. The serum was separated, collected in relabeled tubes, and stored at –20°C. Moreover, all other relevant information about risk factors was recorded over a predesigned questionnaire.

### Detection of the antibodies

All reagent materials were allowed to equilibrate at room temperature (25°C) for 30 min before use. For the detection of antibodies against the VP7 protein, a commercial c-ELISA kit (ID Screen, Grabels, France) was used. The principle of this assay involves competition between the antibodies present in the serum samples and the conjugated antibodies for binding to the antigen-coated plate. The assay outcomes were determined by measuring the absorbance at a wavelength of 450 nm using an ELISA plate reader (Bio-Tec, Vermont, USA), consistent with procedures previously implemented by Sohail *et al*. [10].

### Statistical analysis

Results are expressed as a percentage of signal-to-noise (S/N) ratio using the formula Signal-to-noise percentage = Optical density (OD) value of the sample/Mean OD value of negative control. The S/N value of <70% was considered positive for the presence of anti-VP7 antibodies, as instructed by the manufacturer.

The collected data were entered and analyzed using SPSS version 20 (IBM Corp., NY, USA). The correlation between BTV and different risk factors was determined. Chi-square test was applied with univariate analysis.

## Results

### Prevalence of anti-VP7 antibodies

**Figure-2:**
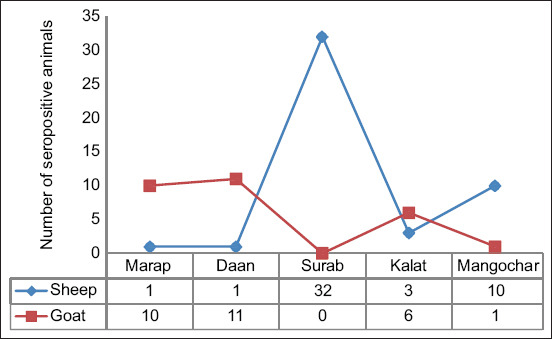
Prevalence of bluetongue disease in Kalat area, Balochistan, Pakistan.

Overall, 27.4% (74/270) of seropositive animals were recorded in the sheep and goat populations of the study area. Animals in Surab City had the highest prevalence of 47% (32/68), followed by Kalat at 28.1% (9/32) and Dan at 24% (12/50) area. However, the lowest prevalence was observed in the Mangochar 15.49% (11/71) and Marap 22.44% (11/49) area (Figure-2).

### Contributory risk factors

In this study, slightly higher seropositivity was observed in female (29%) than male (27%) animals (Table-1). Similarly, a more productive age was seen as more prone to disease, and statistically significant results (confidence interval [CI] = 95%, p = 0.009) were recorded. However, none of the animals under 6 months of age were found positive. However, animals in the 6–36-month age group had the highest prevalence at 39.5% (32/81). In contrast, 22.58% (42/186) seropositivity was seen in ≥36-month age group.

**Table-1:** Relationship between potential risk factors and BTV infection in Kalat, Balochistan.

Risk factors	Number of animals	Number of positive animals	Number of negative animals	Seroprevalence (%)	χ^2^	p-value
Animals	270	74	196	27.4	1.71	0.120
Sheep	150	46	104	30.4		
Goat	120	28	92	23.3		
Age					9.41	0.009
<6 months	3	00	3	0		
6–36 months	81	32	49	39.50		
>36 months	186	42	144	22.58		
Sex					0.025	0.515
Male	28	8	20	28.57		
Female	242	66	176	27.27		
Breed					7.69	0.053
Balochi	145	45	100	31.03		
Rakhshani	6	1	5	16.66		
Khurasani	49	17	32	34.69		
Lehri	70	11	59	15.71		
Grazing style					400.65	0.009
Open	251	00	251	93		
Close	19	00	19	7		
Ticks					0.00	0.800
Yes	57	00	57	21		
No	213	00	213	79		
Area					31.14	0.560
Marap	49	11	38	22.44		
Dan	50	12	38	24		
Surab	68	30	38	44		
Kalat	32	12	20	37.5		
Manguchar	71	9	62	12.67		

BTV=Bluetongue virus

**Figure-3:**
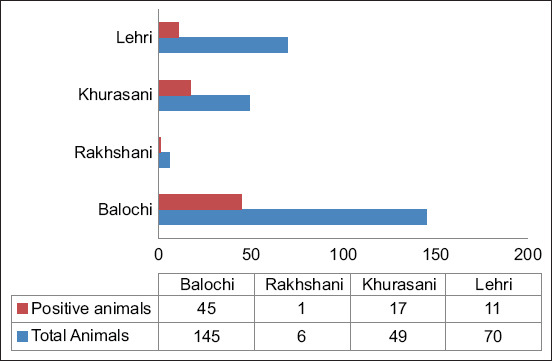
Seropositivity of anti-VP7 antibodies in different breeds of small ruminants in Kalat, Balochistan, Pakistan.

All four different breeds (Balochi, Khurasani, Lehri, and Rakhshani) of the area were found to be seropositive. However, Balochi and Khurasani breed animals were more seropositive than Lehri and Rakhshani (Figure-3).

Almost 93% (CI = 95%; χ^2^ = 400.5, p = 0.009) of animals were grazing in rangelands with mountainous terrain, and only 21% were found infested with external body ticks.

## Discussion

This study aimed to estimate the prevalence of BTV infection in small ruminant population in Kalat, Balochistan, Pakistan. BTV represents a significant economic threat as a vector-borne disease affecting sheep and goats. Various diagnostic methods exist, each with inherent limitations, including prolonged processing times, potential cross-reactivity, and reduced sensitivity. Nevertheless, advancements over the past two decades, particularly in the development of c-ELISA, have markedly improved the efficacy of serological assays, yielding excellent results in the detection and monitoring of the disease [9]. Although, very limited data is available regarding the prevalence of BTV in Pakistan. However, in a recent study from Balochistan [10] comprising four districts, the overall prevalence was 47.26% (414/876, CI, 95%) as compared to 27% (74/271) prevalence using c-ELISA in this study. These findings corroborate with a recent study from four districts of Balochistan, which reported 47.26% prevalence [10]. Similarly, in an earlier study from Khyber Pakhtunkhwa, 44% prevalence was recorded [15]. In contrast, in a recent study from Khyber Pakhtunkhwa, 50% prevalence has been reported [16]. Likewise, in the recent study from Southern Punjab, Pakistan, a very high seroprevalence (70%) was observed in the camel population [17]. All these studies from various provinces of Pakistan indicate an alarming situation. Similarly, a 14% infection rate was reported from Telangana, India [18], 28% in the northeastern part of India [19], and 39.3% in Chattogram, Bangladesh [20]. On the other hand, 47.8% seroprevalence was reported in sheep and goat populations in Jordan [21], 30.3% in southern and 4.7% in northeastern China [22], and 95% in Egypt [23].

Afghanistan and Iran are two neighboring countries of the province, with higher seroprevalence reports from both countries. In a report from Afghanistan, 68.6% sheep, 71.8% goats, and 48.6% cattle were found seropositive using the c-ELISA kit [24]. Similarly, in Iran, 57.59% of sheep, 65.65 % of goats, and 27.63% of cattle have been reported to be seropositive [25]. These reports clearly indicate a serious threat to Pakistan due to the free movement and mass transportation of animals between Pakistan, Afghanistan, and Iran. However, relatively less seropositivity in our study may also be due to the cold climate of the area, and less tick infestation in the animals may have restricted the seroconversion of the disease. Considering the higher specificity and sensitivity (100%, CI = 99.84%–100%) of the c-ELISA kit for BTV, the true picture of seroprevalence is presented in this study. To date, no vaccination is carried throughout Pakistan against BTV infection.

In this study, the prevalence of BTV was found to be slightly higher in sheep at 30.4% compared to 23.3% in goats. However, a recent study from other regions of Balochistan [10] has reported a relatively higher prevalence, with 44% in sheep and 51% in goats. This variation in prevalence rates could be attributed to differences in tick infestation levels among the animal populations studied.

Variable frequencies of seropositivity were observed among different breeds of animals in the study area. Notably, a relatively higher prevalence of infection was found in Balochi and Khurasani breeds compared to Rakhshani and Lehri breeds. Despite these differences, this study indicates that all breeds examined were approximately equally susceptible to infection. This finding contrasts with studies from the Mediterranean Sea and Northern Europe, where animal breeds were reported to have no significant association with differences in seroconversion rates [11].

Almost all age groups in this study were equally susceptible to the disease except early age (<6 months). These findings corroborate with a previous study [10], that almost all age groups are susceptible to infection. In this study, younger animals were found to be uninfected. This might be due to the very low number of samples (3/270) and additionally, older animals have more time (life longevity) to acquire infection from infected animals [26]. Moreover, the increased burden of infection might also be associated with decreased temperature and relative high humidity, which favor the growth of vectors [27, 28].

At present, China has banned the import of ruminants and their related products from Iraq after a report of a bluetongue outbreak by the WOAH [29]. Earlier, China banned imports from the Netherlands and Belgium, as the imported animals (sheep and cows) exhibited mouth ulcers, fever, and bluetongue [29].

## Conclusion

BTV is highly prevalent in small ruminants, both sheep and goat in the study area. Age, sex, and species of the animals were found significantly associated with the disease prevalence. Although this was an exploratory study, further detailed studies are needed to probe the genotype and serotype of the virus circulating in the area with the culicoides involved, which will surely be helpful in devising the disease control strategy in the area.

## Authors’ Contributions

SA and MS: Study design and experimentation. AR: Supervised the laboratory work and facilitation. FB and NUK: Statistical analysis and drafted and reviewed the manuscript. EIA and PRDR-E: Critical review and statistical analysis. HMA and AH: Sample collection and drafted the manuscript. All authors have read and approved the final manuscript.
